# One-year-old wood morphology and colorimetric indicators of cold hardiness in ‘Frontenac’ and ‘Prairie Star’ grapevines

**DOI:** 10.3389/fpls.2026.1843825

**Published:** 2026-06-05

**Authors:** Hava Delavar, Harlene Hatterman-Valenti, Ozkan Kaya

**Affiliations:** 1 Department of Plant Sciences, North Dakota State University, Fargo, ND, United States; 2 Department of Life Sciences, Western Caspian University, Baku, Azerbaijan

**Keywords:** bud survival, low temperature exotherm, phloem, *Vitis* spp., xylem

## Abstract

Cold hardiness is a critical trait for grapevine survival and productivity in cold climates. This study examined the relationships among cane morphological characteristics, shoot color parameters, and cold hardiness in two grapevine cultivars (‘Prairie Star’ and ‘Frontenac’) across four dormant-season sampling times (ST 1–ST 4) and three internode diameter classes (small, normal, and large). Morphological traits, including internode length, shoot diameter, and cross-sectional area, did not show a consistent temporal trend across sampling periods, suggesting that the observed variation was primarily associated with sampling time and cane class rather than progressive structural change during dormancy. In contrast, colorimetric traits showed a clear seasonal pattern, with shoots becoming darker and redder from ST 1 to ST 4, consistent with advancing lignification and cane maturation. Cold hardiness, assessed using low-temperature exotherms of bud, phloem, and xylem tissues, increased substantially from early to mid-dormancy, with xylem tissues reaching the greatest freezing tolerance by ST 3–ST 4. ‘Prairie Star’ showed slightly greater xylem cold hardiness than ‘Frontenac’, while bud survival remained consistently high across all treatments. Strong associations between shoot color and LTE values indicate that color traits, particularly at the fifth internode, may serve as reliable non-destructive indicators of cold hardiness status. Sampling time was the primary source of multivariate variation, with cultivar and internode class contributing secondary effects. These findings demonstrate that observable cane traits, especially shoot color, reflect the progression of seasonal cold acclimation and may support the evaluation and selection of cold-hardy grapevine germplasm.

## 1 Introduction

Temperature is one of the fundamental environmental factors affecting the growth of grapevines, just as it is for other crops, and therefore it plays a key role in the regional distribution and cultivation potential of any given, species, genotype or/and cultivar (Kaya and Kose, 2020; Kaya et al., 2024). Considering that grapevines (*Vitis* spp.) are a perennial crop cultivated under many climatic conditions, in regions experiencing severe winter temperatures, extreme temperature events inflict significant damage to canes and buds, affecting not only the survival of the vineyards but also their productivity to a considerable extent (Kaya and Köse, 2017; Kose et al., 2024a). Especially, in these areas with stress factors that push the limits of economically viable cultivation, low-temperature damages below the freezing point can inflict significant losses on grape growers, wholesalers, processing plants, and industrial facilities relying on grape production (Kose et al., 2024b). As known, the European grapevine *Vitis vinifera*, which is widely cultivated worldwide, is susceptible to cold and not well-suited for grape production in the northern mid-section of the United States. It has, indeed, been reported that major freezing events experienced in the eastern United States since 2007, where *Vitis vinifera* varieties are cultivated, have caused economic damages averaging around $250 million annually for the grapevine industry (Poni et al., 2022). For these regions, it is generally preferred to cultivate wild North American grapevine species that are adapted to the local conditions. Species such as *Vitis labrusca* and *Vitis riparia* can survive extremely low winter temperatures, ranging from -35 to -40 °C. As a matter of fact, the survival of buds in grapevines during the winter period is crucial for grape production in the following growing season. These buds form during the summer months and must remain dormant and develop cold hardiness throughout the fall to survive low temperatures in winter before resuming growth and development in spring (Kovaleski et al., 2018). The ability to predict primary bud cold hardiness would enable growers to monitor conditions and decide when it is necessary to evaluate winter bud injury, particularly in currently cold growing regions. Accurate predictions can also be used to assess the current suitability for expansion into other areas or to identify changes in regional suitability according to climate change. Dormancy establishment in grapevines typically occurs in response to shortening photoperiod in late summer and fall (Fennell and Hoover, 1991), but other environmental factors also play a role (Shellie et al., 2018). The advancement of dormancy is subsequently regulated by the exposure to chilling temperatures, whereby the plant undergoes a transition from a stage unresponsive to warm temperatures (endodormancy) to a phase that is responsive to such conditions (eco-dormancy). The release from this eco-dormant state occurs when the vine is subjected to the rise of warm temperatures during the spring season (Lang et al., 1985).

Cold hardiness usually exhibits a U-shaped pattern with three distinct stages during the progression from fall to winter to spring (Ferguson et al., 2014). In the fall, acclimation leads to gains in cold hardiness of grapevine buds through the establishment and enhancement of supercooling. In mid-winter, buds reach maximum cold hardiness and maintain high levels of cold hardiness depending on environmental cues. Finally, in late winter and early spring, as warm temperatures return, de-acclimation leads to a loss of cold hardiness, although re-acclimation is possible if low temperatures return (Kovaleski et al., 2018). In regions prone to severe cold spells, the renewal of grapevine trunks damaged by freezing temperatures is a crucial practice for viticulture. In North Dakota viticulture, the snow cover protects grapevine trunks up to 5–10 cm above ground level. In spring, new cordons are formed from these trunks, and grapes are harvested from the shoots on these cordons. Although this practice is implemented in this region, growers often rely on anecdotal information due to the absence of published research on grapevine renewal strategies from a viticultural perspective (Santos et al., 2020). In such regions, determining the critical temperatures of shoots and buds of grape cultivars or genotypes can play a pivotal role in developing novel approaches and strategies aimed at addressing the current challenge. In this regard, differential thermal analysis (DTA) is a widely employed technique for assessing frost tolerance and cold hardiness in buds, flowers, and canes of various deciduous woody species, including grapevines (Andrews et al., 1984; Kaya et al., 2018, 2020; 2021a, 2021b, 2021c; Kose and Kaya, 2022; Kaya and Kose, 2022a, 2022b). This method systematically identifies thermal effects and freezing processes in and around buds by subjecting samples to variable temperature cycles, allowing for the determination of the latent heat of fusion (Kaya et al., 2021c). DTA distinguishes the latent heat of fusion as exothermic high-temperature (HTE) and low-temperature (LTE) events, resulting from the crystallization of extracellular and intracellular supercooled water, respectively, during exposure to a controlled temperature program (Andrews et al., 1984). While previous research has primarily focused on the critical temperatures of grapevine shoots and dormant buds, there is currently a limited understanding of the relationship between shoot morphology and a grapevine’s ability to withstand low winter temperatures. Some scholars have suggested that shoot morphology and anatomy may possess significant characteristics that influence cold hardiness, where larger diameter (12–15 mm) canes were shown to be more sensitive to cold than their normal counterparts (7–9 mm diameter) (Todaro and Dami, 2017). Furthermore, Köse and Güleryüz (2009) reported a significant negative correlation (r = −0.726) between one-year-old shoot diameter, categorized into three classes (6.0–8.0, 8.1–10.0, and 10.1–12.0 mm), and the low-temperature tolerance of primary buds during winter dormancy. Consequently, developing a meaningful explanation of the relationship between the morphology and cold hardiness of these one-year-old shoots, which arose from damaged trunks, could provide a valuable approach to trunk renewal strategies. From this perspective, visual characteristics such as shoot diameter and shoot color may serve as useful indicators in determining the potential and survival probability (LTE values) of selected shoots.

Despite the pivotal role of grapevine shoot morphology in determining cold hardiness and its potential implications for trunk renewal strategies, there is currently a paucity of research addressing this critical aspect of viticulture. To help bridge this knowledge gap, a study was conducted on reestablished cordons after winter temperatures severely injured vine trunks with the following objectives: (i) to characterize the cold hardiness of two grapevine genotypes throughout the winter season, focusing on two widely cultivated genotypes in North Dakota, (ii) to elucidate the relationship between cane color, diameter, and cold hardiness in one-year-old shoots and dormant buds, thereby providing valuable insights into the complex interplay of morphological traits and low-temperature hardiness. By investigating these physiological mechanisms during the dormant period, this study aims to characterize the complex factors governing grapevine shoot cold hardiness, providing a framework for subsequent viticultural research. The knowledge gained from study will not only aid grapevine breeders in developing novel cultivars with enhanced cold hardiness but also empower growers to optimize their vineyard management practices, ultimately ensuring the long-term sustainability and prosperity of the viticulture industry in the face of increasingly challenging environmental conditions.

## 2 Materials and methods

### 2.1 Plant materials

The study was conducted at the North Dakota State University Horticulture Research Farm (NDSU-HRF) near Absaraka, ND, USA, in Cass County (46°99’065.7”N, 97°35’581.1”W, 315 m elevation). The site falls within USDA Plant Hardiness Zone 4a (USDA-ARS, 2012). Two adjacent experimental vineyards were used: a ‘Frontenac’ block planted in 2006 and a ‘Prairie Star’ block planted in 2009. Both vineyards were own-rooted and non-irrigated, with a planting layout of 2.5 m between rows and 3 m between vines within rows. Vineyard management followed standard regional practices for cold-climate viticulture in North Dakota, including routine fertilization, weed control, canopy management, and dormant-season spur pruning; no supplemental irrigation was applied during the experiment.

The cold-hardiness measurements reported here were carried out during the 2023–24 dormant season on vines that had been re-established following severe winter injury. During the 2018–2019 dormant season, an extreme cold event killed the majority of the above-ground plant material at the NDSU-HRF, including substantial trunk death up to 10–15 cm above the soil line on most research vines. To re-establish the ‘Frontenac’ and ‘Prairie Star’ plots, approximately four healthy suckers per vine position were retained during the 2019 and 2020 growing seasons, tied to a vertical training pole, and re-tied every two to four weeks throughout the active growth period to maintain a vertical orientation until cordon establishment. The experimental period of the present study, the retrained vines had developed a stable bilateral cordon structure; of the four initial cordons established during retraining, two three-year-old cordons were retained per vine, collectively carrying 16–18 spur-pruned canes.This dual-cultivar setting of two own-rooted, non-irrigated cultivars sharing the same retraining history, soil, and microclimate, provided a directly comparable framework for evaluating the genotype effect on cold hardiness. The experiment was laid out as a randomized complete block design (RCBD) with nine blocks (replicates) per genotype. Each vineyard was subdivided into nine contiguous blocks oriented perpendicular to the prevailing slope and the row direction, so that each block captured comparable microenvironmental variation (slope, soil moisture, exposure). Within each block, six adjacent vines of the same genotype constituted one experimental unit; thus, each genotype was represented by 9 blocks × 6 vines = 54 vines in total. Vines exhibiting visible disease symptoms, mechanical damage, or atypical vigor were excluded prior to randomization. At each sampling date, two healthy, sun-exposed, well-lignified one-year-old shoots per diameter class (small, normal, larger) were collected from each experimental unit, yielding 9 shoots × 3 classes × 2 genotypes = 54 shoots per sampling date.

For the univariate analyses (ANOVA), the full nine-block dataset was retained whenever an observation was available; only blocks with complete observations across all four sampling dates and all three diameter classes were retained, resulting in six fully balanced blocks. The selection criteria for one-year-old shoots included health (indicated by dark-brown periderm sections), full sun exposure, diameter class (small, 6.0–8.0 mm; normal, 8.1–10.0 mm; larger, 10.1–12.0 mm), and the presence of well-developed periderm on at least eight internodes. Considering the established understanding that lignification in grapevine canes normally begins at the basal bud and advances towards the apical bud, the sampling and measurement protocol was designed to account for this acropetal gradient in tissue maturity and hardiness (Fennell, 2004). Shoots were collected at four time points across the dormant season: the third week of October (ST 1), November (ST 2), December (ST 3), and February (ST 4). At each sampling date, collections were made two hours after sunrise to minimize diurnal environmental variation. Samples were immediately placed in sealed polyethylene bags to prevent desiccation, transported to the laboratory, and processed on the same day. Climatic data were obtained from the North Dakota Agricultural Weather Network (NDAWN; https://ndawn.ndsu.nodak.edu/weather-data-daily.html). A summary of the meteorological events recorded between September 2023 and January 2024, daily mean, minimum, and maximum air temperatures; cumulative chilling hours below 7.2 °C; absolute minimum-temperature events; and precipitation, is presented in NDAWN.

### 2.2 Morphological characterization of one-year-old canes

#### 2.2.1 Internode measurements

In the laboratory, the total number of lignified internodes on each cane was visually counted. Bud positions were numbered following the standard viticultural convention in which “bud 1” denoted the first count bud distal to the basal compressed zone (i.e., distal to the non-count basal bud/bourrelet). Accordingly, IL_1_ was defined as the internode between count bud 1 and count bud 2, IL_2_ as the internode between buds 2 and 3, and so on up to IL_5_ between buds 5 and 6 (Smart and Robinson, 1991; Keller, 2010). To standardize measurements, the six basal count nodes (buds 1–6) were retained by trimming three to eight nodes distal to the sixth node, depending on cane vigor. Internode length was measured for all five basal internodes (IL_1_ through IL_5_) using a digital caliper with 0.01 mm resolution. Winter hardiness and primary low-temperature response were assessed following the internode measurement protocol described by Svyantek et al. (2023). For internodes one and five, representing the basal and distal ends of the measurement zone, the following parameters were quantified:

Internode Length (IL): linear distance (mm) between the two adjacent nodes delimiting each internode, measured along the cane axis with the caliper jaws aligned with the proximal and distal node scars. Because IL_1_ corresponds to the basal compressed internode, its length is characteristically short, generally in the range of 10–25 mm, and was therefore measured with additional care to exclude the underlying basal bourrelet, which would otherwise lead to overestimation of IL_1_ (Pratt, 1974; Keller, 2010).
Maximum Diameter: greatest caliper measurement across the internode (mm).
Minimum Diameter: smallest caliper measurement perpendicular to the maximum diameter (mm).

The following secondary parameters were calculated from the primary measurements:

Average Caliper Diameter (CAL) = (Maximum Diameter + Minimum Diameter)/2.
Cane Diameter Ratio (CDR) = Maximum Diameter/Minimum Diameter.
Cane Internode Diameter Ratio (CIDR) = Internode Length/CAL.

Cross-sectional cane areas (CCA_1_ and CCA_5_) were calculated from the average caliper diameter assuming a circular geometry, using the formula CCA = π × (d/2)^2^, where *d* represents the measured average diameter (mm) at the 1st and 5th internodes; results were expressed in mm^2^.

#### 2.2.2 Color analysis

**Figure 1:**
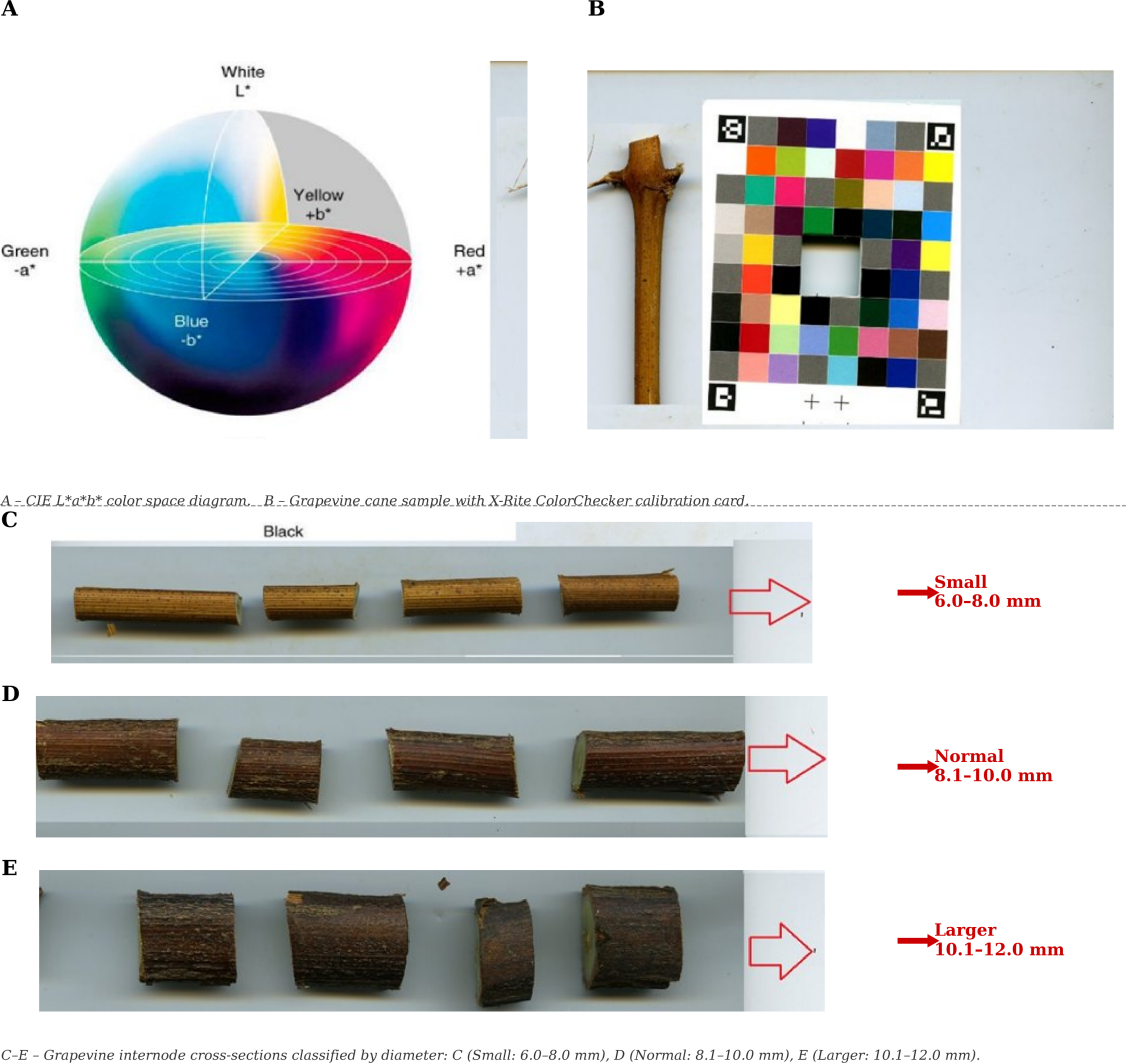
CIE L*a*b* color space used for colorimetric measurements on one-year-old grapevine shoots **(A)**, representative shoot sample positioned alongside an X-Rite ColorChecker calibration card for standardized color acquisition **(B)**, and cross-sectional views of grapevine internodes classified into three diameter classes: small (6.0–8.0 mm) **(C)**, normal (8.1–10.0 mm) **(D)**, and larger (10.1–12.0 mm) **(E)**. Color parameters L* (lightness), a* (red–green axis), and b* (yellow–blue axis) were measured according to Cortez et al. (2017).

In addition to the morphological measurements, subsamples were excised from internodes one and five of each cane for color analysis. Because IL_1_ is intrinsically short (typically 10–25 mm), the section excised from this position consisted of the entire IL_1_ segment together with a short portion (≤5 mm) of the adjacent nodes, whereas from internode 5 a section of approximately 100 mm was excised, centered on IL_5_ (**Figure 1F**). Sections were scanned at 600 dpi using a flatbed scanner (**Figure 1B**). All images were captured with the same scanner (Epson Perfection V600, Seiko Epson Corp., Suwa, Japan under identical acquisition settings (resolution, gamma, white balance) throughout the entire study. To ensure inter-session comparability, color calibration was performed at the beginning of every scanning session against the X-Rite ColorChecker Classic card shown in **Figure 1B**, and the resulting ICC profile was applied to all images of that session prior to analysis (Cortez et al., 2017). The resulting image files were analyzed using ImageJ software.

Within ImageJ, the RGB (red, green, blue) color values were measured for each pixel and converted to CIELAB color space coordinates (L* for lightness, a* for green–red, and b* for blue–yellow). CIELAB color space allowed color to be represented in a perceptually uniform manner, providing a quantitative assessment of visible color differences (Cortez et al., 2017). For each scanned section, a rectangular region of interest (ROI) was defined on the periderm surface, excluding shadows, scanner edges, and the node scar areas, and pixel-wise CIELAB values within the ROI were averaged.

The following color parameters were quantified:

L_mean: mean L* value representing overall lightness/darkness.
a_mean: mean a* value representing the green (negative) to red (positive) component.
b_mean: mean b* value representing the blue (negative) to yellow (positive) component.
ΔE: total color difference from a standard white reference tile (ΔE = √[(L_mean)^2^ + (a_mean)^2^ + (b_mean)^2^]).

Chroma (C*) and hue angle (h°) were additionally derived as C* = √(a*^2^ + b*^2^) and h° = arctan(b*/a*), respectively, and reported separately for IL_1_ (C_1_, h°_1_, L_1_, a_1_, b_1_) and IL_5_ (C_5_, h°_5_, L_5_, a_5_, b_5_).

#### 2.2.3 Evaluation of cold hardiness of the buds and cane parts with DTA

The principal assessment of bud, xylem and phloem cold hardiness, inclusive of low-temperature exotherms (LTE), was executed employing DTA, as delineated by Kaya and Kose (2020). Buds ranging from the 1^st^ to the 6^th^ node (1^st^, 2^nd^, 3^rd^, 4^th^, 5^th^, and 6^th^) were excised, with care taken to preserve approximately 2 mm of underlying woody tissue intact to ensure consistent and accurate assessment of true bud mortality in the field, following Mills et al. (2006). One-year-old shoots were cut to 4–6 mm thickness and these shoots belonging to the first 5 nodes were tested. Then, these buds and cane parts were positioned on a thermo-electric module (TEM) within a Tenney Junior Environmental Test Chamber (model T2C-A -F4T, Thermal Product Solutions, New Columbia, PA), which was outfitted with a temperature controller (Test Equity LLC 6100 Condor Drive Moorpark, CA 93021). In the experimental setup, buds and cane parts from the 1^st^ to the 6^th^ nodes were systematically placed in corresponding wells on TEM trays. The study was designed to include nine replicates, with each replicate comprising six buds, thereby, analyzing 54 buds and canes from each node for each grape variety at each sampling interval. The freezer chamber’s protocol commenced with a stabilization phase at 4 °C for 1 hour, followed by a controlled decrease in temperature from 4 °C to -44 °C at a decrement rate of 4 °C per hour. For each DTA assessment, the electrical voltage output from the TEMs, measured in millivolts (mV), was recorded on a computer. This analysis facilitated the calculation of the critical temperatures at which 10%, 50%, the average, and 90% of the dormant buds and cane parts were lethally affected by cold, denoted as LTE_10_, LTE_50_, and LTE_90_, respectively. These critical temperature thresholds were established following the methodology outlined by Kaya and Kose (2020), providing a quantifiable measure of the cold hardiness of the grapevine buds and cane parts under study. Also, after each sampling date, an additional set of six buds per node per experimental unit (separate from those used for DTA) was retained and incubated to confirm viability *in vivo*. Bud viability was assessed using the standard transverse-section method (Wolpert and Howell, 1985): each compound bud was cross-sectioned with a razor blade approximately at its mid-point, and the primary, secondary, and tertiary meristems were inspected under a stereomicroscope (10–25× magnification). Buds whose primary meristem appeared bright green and turgid were scored as alive; those exhibiting brown, water-soaked, or necrotic tissue were scored as dead.

### 2.3 Statistical analysis

All LTE values obtained from the DTA were presented as mean ± standard error (SE), derived from nine independent replicates to ensure the reliability and reproducibility of the data. In the context of evaluating the hardiness of grapevine buds and cane parts to cold stress, the following variables were defined to model the adjustments in cold hardiness levels due to varying degrees of temperature-induced stress:

Xa was designated to represent the baseline physiological metric of the grapevine buds and cane parts, (e.g., maximum cold hardiness level or lowest LTE value).

Xb quantified the magnitude of change in this baseline metric resulting from exposure to different cold temperature conditions, or it is the difference between the maximum and minimum LTE values.

The percentages -10%, 50%, and 90%- were interpreted as indicative of minor, moderate, and severe damage, or damage levels, respectively, caused by these temperature variations.

CHbase represented the baseline cold hardiness level of grapevine buds and cane parts (corresponding to Xa).

ΔCH represented the change in cold hardiness due to cold stress (corresponding to Xb).

CHadjusted represented the adjusted cold hardiness level after exposure to cold stress.

Accordingly, the adjusted cold hardiness levels post-exposure to cold stress were modeled using the following formulations:

Minor Stress Impact: The adjusted cold hardiness level after a minor damage impact, calculated as 10% of the change induced by the cold stress, was given by:

CHadjusted = CHbase - (0.1 × ΔCH*)*.

This formula aimed to quantify the hardiness of the buds and cane parts when subjected to minimal cold stress, providing insights into their initial response to cold temperatures.

Moderate Stress Impact: The cold hardiness level following a moderate stress impact, accounting for 50% of the induced change, was determined as:

CHadjusted = CHbase - (0.5 × ΔCH*)*.

This calculation helped to understand the mid-range adaptability of grapevine buds and cane parts to increasing levels of cold damage.

Severe Stress Impact: The cold hardiness level in the wake of a severe damage impact, reflecting 90% of the change due to cold exposure, was assessed with:

CHadjusted = CHbase - (0.9 × ΔCH*)*.

This calculation was critical for evaluating the upper limits of the grapevine cane parts and buds’ hardiness to extreme cold conditions. All descriptive analyses were carried out utilizing the agricolae package in R Studio (Mendiburu, 2023). The full statistical model used to evaluate the effects of sampling time, genotype, and diameter class on each morphological, color, and LTE variable was a linear mixed-effects model of the form:

Y_ijklm = μ + ST_i + G_j + C_k + (ST × G)_ij + (ST × C)_ik + (G × C)_jk + (ST × G × C)_ijk + Block_l + (Block × ST)_li + ϵ_ijklm.

where Y_ijklm is the response variable, μ is the overall mean, ST_i is the fixed effect of sampling time (i = 1–4), G_j is the fixed effect of genotype (j = Frontenac, Prairie Star), C_k is the fixed effect of diameter class (k = small, normal, larger), Block_l is the random effect of block (l = 1–9, capturing spatial heterogeneity in the field), (Block × ST)_li is the random block-by-sampling-time interaction (capturing the temporally repeated structure of measurements on the same block), and ϵ_ijklm is the residual error.

Because sampling time and node/internode position are not randomly distributed but ordered along temporal and morphological axes, autocorrelation among repeated measurements was explicitly modelled. For variables measured repeatedly on the same block across the four sampling dates, an AR(1) (first-order autoregressive) covariance structure was specified for the residuals using the nlme package (Pinheiro et al., 2023); for variables structured along node/internode position, a corCAR1 continuous-time autoregressive structure indexed by node number was tested. The covariance structure that minimized AIC was retained for each variable. Normality and homoscedasticity of residuals were checked using Shapiro–Wilk tests and diagnostic residual plots; when assumptions were violated, response variables were transformed (log or Box–Cox) before re-fitting.

Fixed effects were tested by Type-III ANOVA. *Post-hoc* comparisons among means within significant interactions were performed by Tukey HSD via the agricolae package, with α = 0.05. Prior to Principal Component Analysis (PCA), all variables were centered and scaled to unit variance (mean = 0, SD = 1) using the scale function in base R, so that variables measured on different units (mm, mm^2^, °C, dimensionless ratios, CIELAB units) contributed equally to component construction. Missing values were excluded list-wise. PCA was carried out using ggbiplot in R Studio (Wickham, 2016). The heatmap was conducted via the pheatmap package in R Studio (Kolde, 2019).

## 3 Results

### 3.1 Morphological parameters of one-year-old shoots

**Figure 2:**
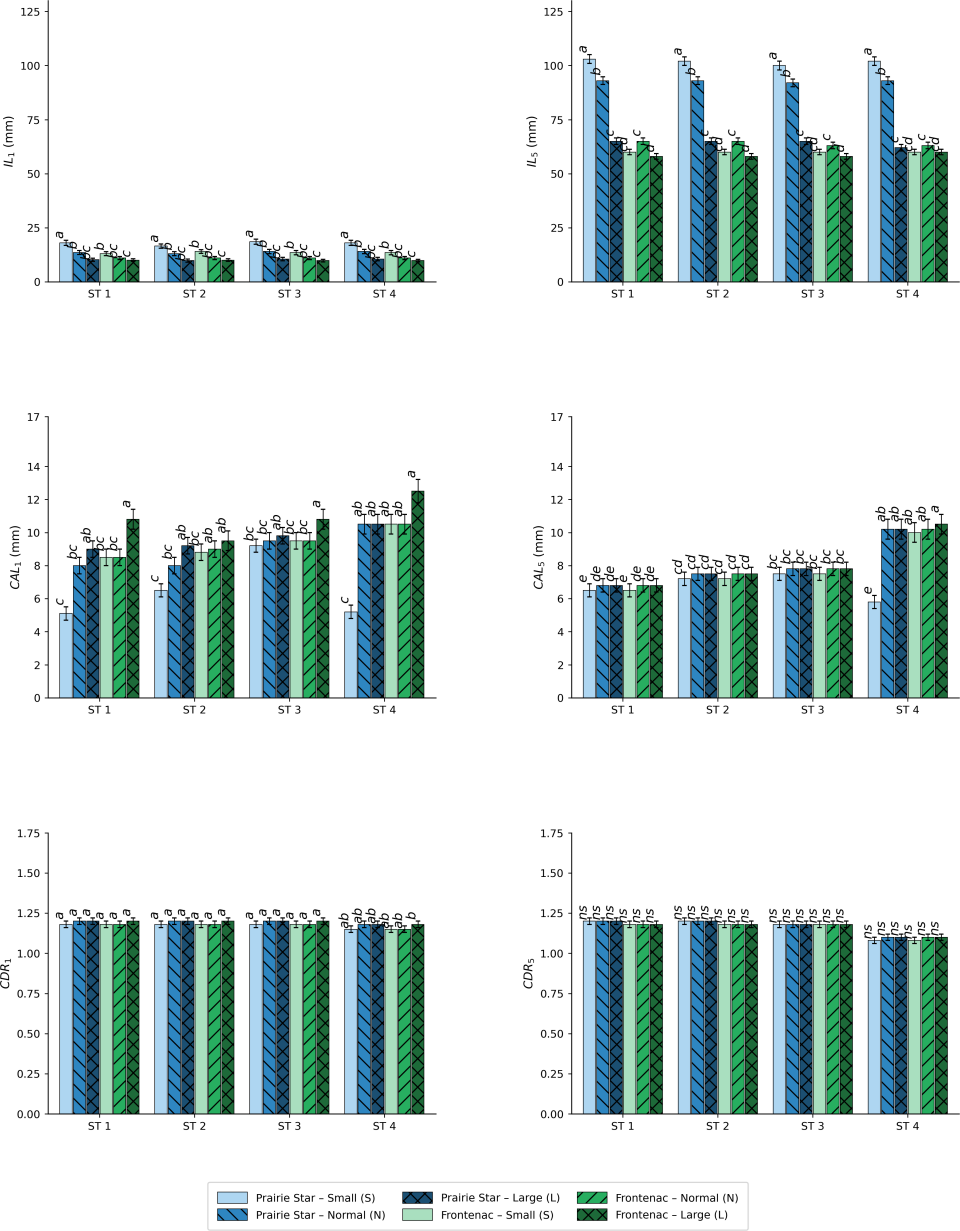
Mean values (± SE) of IL_1_, IL_5_, CAL_1_, CAL_5_, CDR_1_ and CDR_5_ in ‘Prairie Star’ and ‘Frontenac’ grapevine genotypes under three internode classes (S: small, N: normal, L: large) across four sampling times (ST 1–4). Each mean was calculated from 54 independent measurements (9 blocks × 6 vines per block). Different lowercase letters indicate significant differences according to the Sampling time (ST) × Genotype (G) × Class (C) interaction (*p* < 0.05). Abbreviations: CAL_1; average diameter for internode 1, CAL_5; average diameter for internode 5, CDR_1; cane diameter ratio for internode 1, CDR_5; cane diameter ratio for internode 5, IL_1; internode length for internode 1, IL_5; internode length for internode 5.

**Figure 3:**
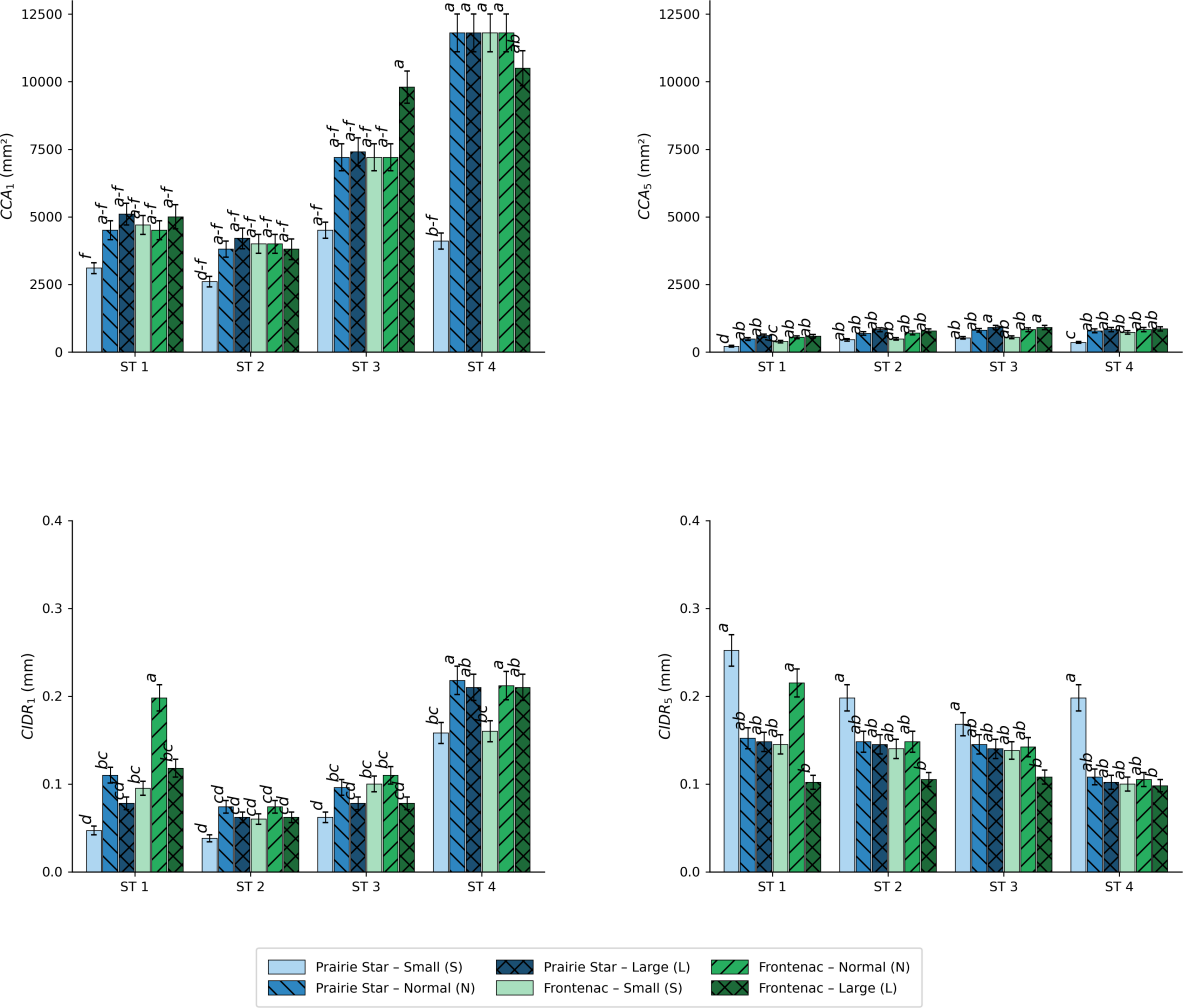
Mean values (± SE) of CCA_1_, CCA_5_, CIDR_1_ and CIDR_5_ in ‘Prairie Star’ and ‘Frontenac’ grapevine genotypes under three internode classes (S: small, N: normal, L: large) across four sampling times (ST 1–4). Each mean was calculated from 54 independent measurements (9 blocks × 6 vines per block). Different lowercase letters indicate significant differences according to the Sampling time (ST) × Genotype (G) × Class (C) interaction (*p* < 0.05). Abbreviations: CCA_1; cross-sectional cane area for internode 1, CCA_5; cross-sectional cane area for internode 5, CIDR_1; cane internode to diameter ratio for internode 1, CIDR_5; cane internode to diameter ratio for internode 5.

Internode length at the first node (IL_1_) ranged approximately between 10–20 mm, with ‘Prairie Star’ generally exhibiting higher values (≈15–18 mm) than ‘Frontenac’ (≈10–14 mm) (**Figure 2**). Across diameter classes, small and normal shoots tended to show slightly higher IL_1_ values than the large class. No consistent temporal trend was observed across sampling times (ST1–ST4) (**Figure 2**). At the fifth node (IL_5_), values varied between ~58 and 105 mm, with ‘Prairie Star’ (≈95–102 mm) consistently exceeding ‘Frontenac’ (≈58–65 mm) (**Figure 2**). Differences among diameter classes were relatively limited, and temporal variation remained weak (**Figure 2**). Shoot diameter at the first node (CAL_1_) ranged from approximately 5.0 to 12.5 mm, increasing from ≈5–9 mm (ST1) to ≈10–12.5 mm (ST4) (**Figure 2**). ‘Frontenac’ generally showed higher values than ‘Prairie Star’, and a clear class effect was evident (L > N > S) (**Figure 2**). A similar pattern was observed for CAL_5_, which ranged between ~6.0 and 13.0 mm and increased progressively toward ST4 (**Figure 2**). Cane diameter ratio values showed minimal variation, with CDR_1_ ranging between 1.15–1.22 and CDR_5_ between 1.08–1.20, without consistent differences among genotypes, classes, or sampling times (**Figure 2**). Cross-sectional cane area at position 1 (CCA_1_) increased markedly over time, from approximately 3000–5000 mm^2^ (ST1) to 10,000–12,500 mm^2^ (ST4) (**Figure 3**). Large-class shoots consistently exhibited the highest values, followed by normal and small classes (**Figure 3**). At position 5, CCA_5_ ranged between ~500 and 1,100 mm^2^, also showing a gradual increase across sampling times (**Figure 3**). CIDR_1_ values ranged from ~0.04 to 0.22, with higher values generally observed at later sampling times (ST4), particularly in normal and large classes (**Figure 3**). CIDR_5_ ranged between 0.10 and 0.25, with small-class shoots occasionally showing higher values, although this pattern was not consistent (**Figure 3**).

### 3.2 Shoot color parameters

**Figure 4:**
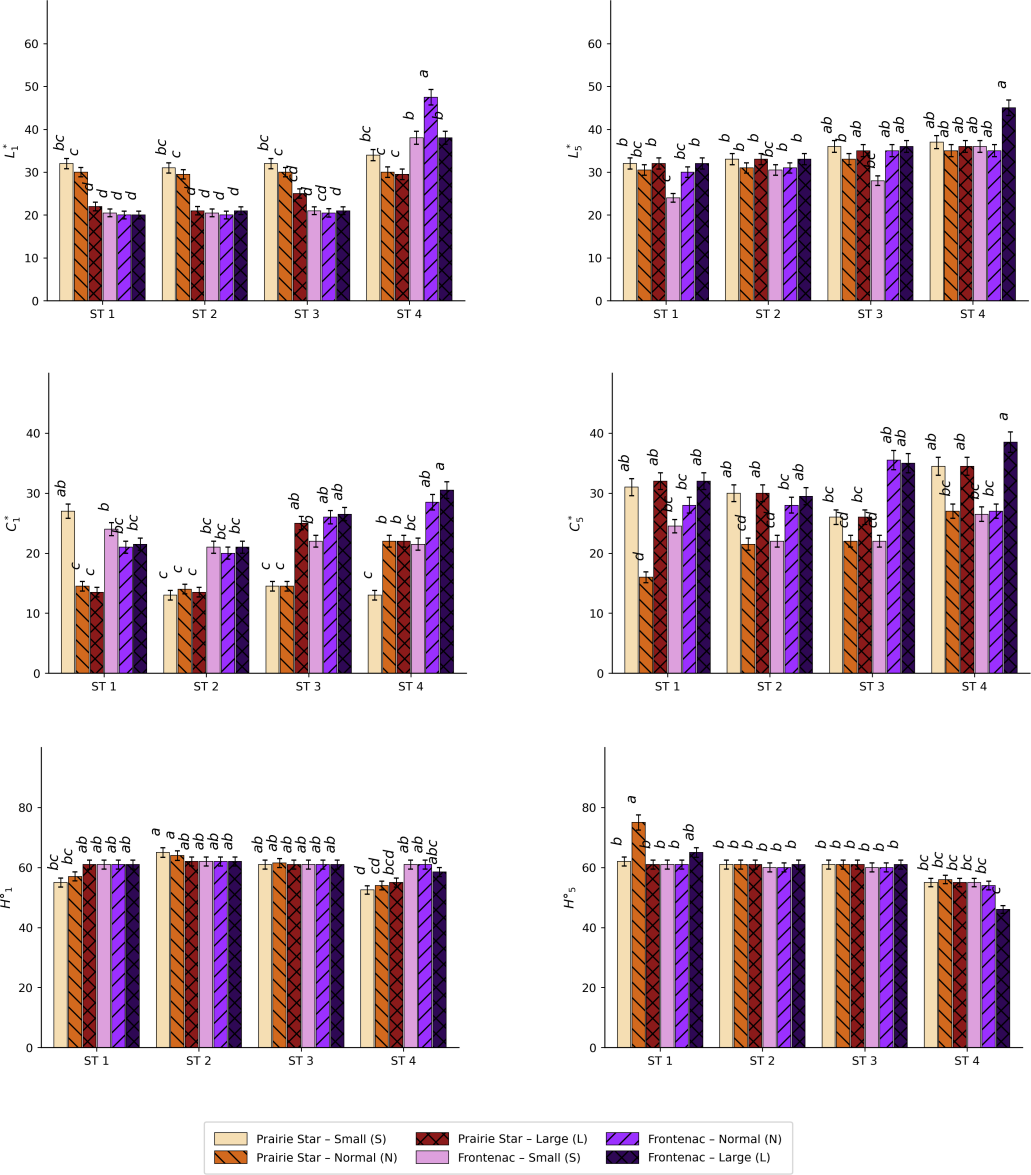
Mean values (± SE) of L*_1_, L*_5_, C*_1_, C*_5_, H°_1_ and H°_5_ in ‘Prairie Star’ and ‘Frontenac’ grapevine genotypes under three internode classes (S: small, N: normal, L: large) across four sampling times (ST 1–4). Each mean was calculated from 54 independent measurements (9 blocks × 6 vines per block). Different lowercase letters indicate significant differences according to the Sampling time (ST) × Genotype (G) × Class (C) interaction (*p* < 0.05). Abbreviations: C*_1_*; C* (chroma) for internode 1, *C*_5_; C* (chroma) for internode 5, H°_1_; h° (hue angle) for internode 1, H°_5_; h° (hue angle) for internode 5, L*_1_*; L* (lightness) for internode 1, L*_5_*; L* (lightness) for internode 5.

**Figure 5:**
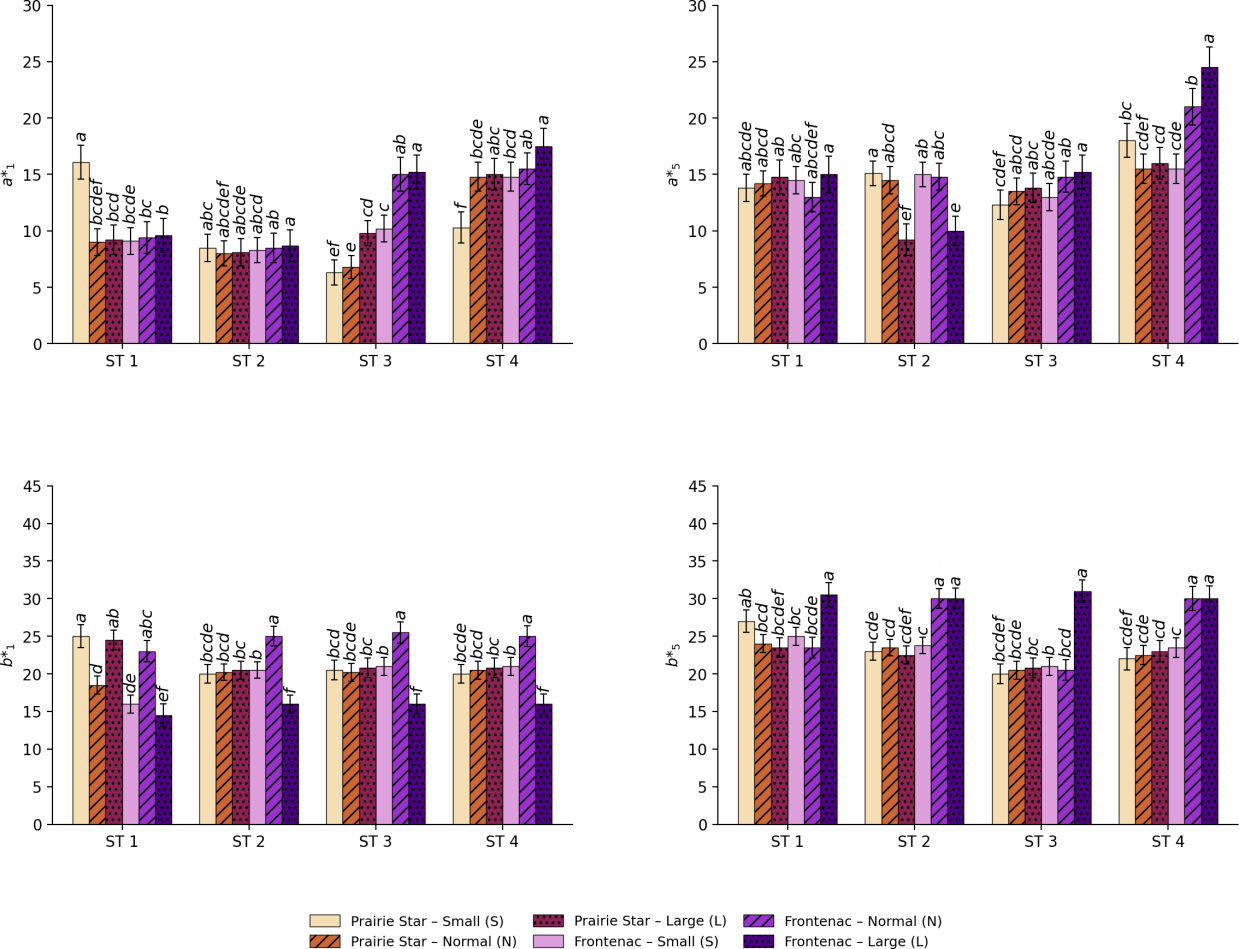
Mean values (± SE) of a*_1_, a*_5_, b*_1_ and b*_5_ in ‘Prairie Star’ and ‘Frontenac’ grapevine genotypes under three internode classes (S: small, N: normal, L: large) across four sampling times (ST 1–4). Each mean was calculated from 54 independent measurements (9 blocks × 6 vines per block). Different lowercase letters indicate significant differences according to the Sampling time (ST) × Genotype (G) × Class (C) interaction (*p* < 0.05). Abbreviations: a*_1_*; a*(green-red) for internode 1, *a*_5_; a*(green-red) for internode 5, b*_1_*; b*(blue-yellow) for internode 1, *b*_5_; b*(blue-yellow) for internode 5.

Lightness (L*) exhibited a clear temporal trend at both internode positions, reaching minimum values at ST 2 (L_1_: 26.3; L_5_: 24.5) and maximum values at ST 4 (L_1_: 45.0; L_5_: 41.4), while ST 1 and ST 3 showed intermediate and comparable levels (**Figure 4**). Differences between cultivars (‘Prairie Star’: 36.9; ‘Frontenac’: 35.1) and diameter classes were minimal. Chroma (C*) at internode 1 increased steadily from ST 1 (17.0) to ST 4 (27.0), whereas at internode 5 it peaked at ST 3 (31.8) and slightly declined at ST 4 (29.3) (**Figure 4**). Variation among cultivars and diameter classes remained negligible. Hue angle (H°) decreased consistently over time at both positions (H°_1_: 66.2° → 53.9°; H°_5_: 70.4° → 52.1°), indicating a progressive shift toward redder–browner coloration, with no meaningful cultivar effect (**Figure 4**). The a* parameter showed the strongest temporal response, increasing markedly from ST 1 to ST 4 at both positions, with slightly higher values observed in large-diameter shoots and marginally higher a_5_ values in ‘Frontenac’ (**Figure 5**). Similarly, b* values increased over time; however, at internode 5 the maximum was recorded at ST 3 (28.8) rather than ST 4 (27.2), and differences among diameter classes were limited (**Figure 5**).

### 3.3 Low temperature exotherms and bud survival

**Figure 6:**
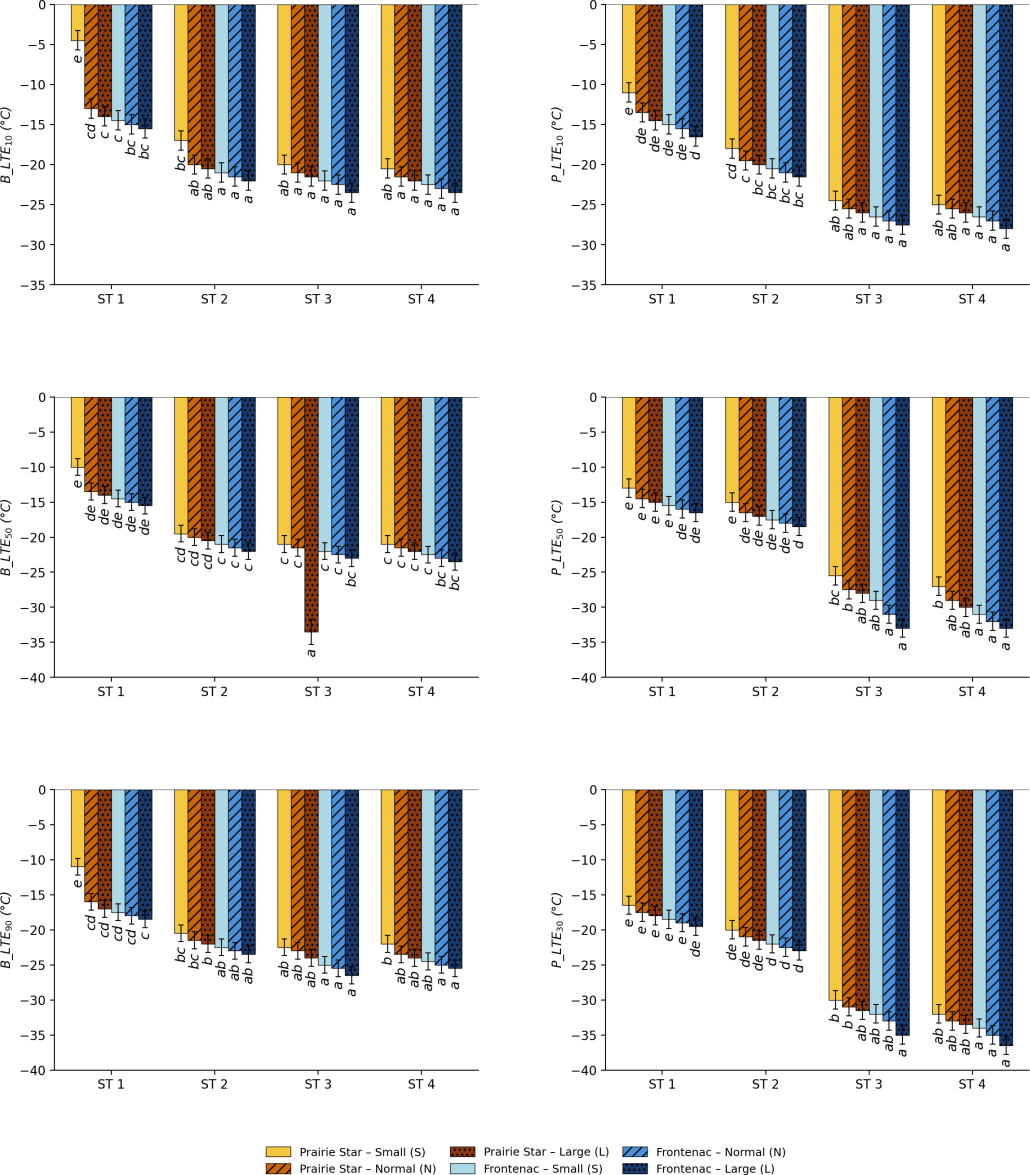
Mean values (± SE) of B_LTE_10_, B_LTE_50_, B_LTE_90_, P_LTE_10_, P_LTE_50_ and P_LTE_90_ (°C) in ‘Prairie Star’ and ‘Frontenac’ grapevine genotypes under three internode classes (S: small, N: normal, L: large) across four sampling times (ST 1–4). Each mean was calculated from 54 independent measurements (9 blocks × 6 vines per block). Different lowercase letters indicate significant differences according to the Sampling time (ST) × Genotype (G) × Class (C) interaction (*p* < 0.05). Abbreviations: B_LTE_10/50/90_; bud LTE_10/50/90_, P_LTE_10/50/90_; phloem LTE_10/50/90_.

**Figure 7:**
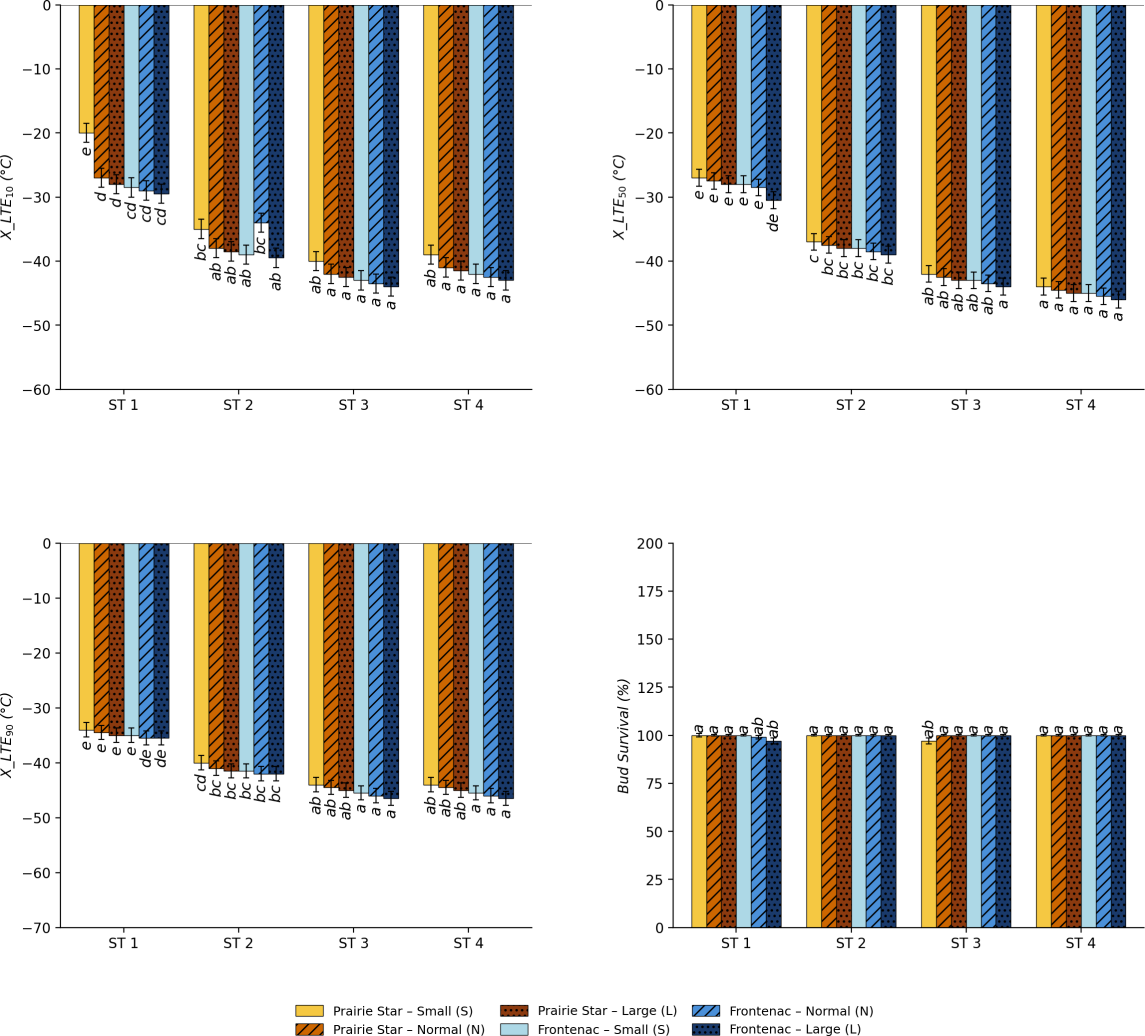
Mean values (± SE) of X_LTE_10_, X_LTE_50_, X_LTE_90_ (°C) and bud survival (%) in ‘prairie star’ and ‘frontenac’ grapevine genotypes under three internode classes (S: small, N: normal, L: large) across four sampling times (ST 1–4). Each mean was calculated from 54 independent measurements (9 blocks × 6 vines per block). Different lowercase letters indicate significant differences according to the Sampling time (ST) × Genotype (G) × Class (C) interaction (*p* < 0.05). Abbreviations: X_LTE_10/50/90_; xylem LTE_10/50/90_, bud_survival; percent of buds 1 to 6 alive at sampling.

Bud low-temperature exotherms (B_LTE) showed a general decline toward more negative values from ST 1 to ST 3, with values at ST 4 remaining at a similar level to ST 3 (**Figure 6**). B_LTE_10_ ranged from approximately −5 to −12 °C at ST 1, −16 to −20 °C at ST 2, −20 to −23 °C at ST 3, and −18 to −22 °C at ST 4. B_LTE_50_ followed a comparable pattern (ST 1: −10 to −15 °C; ST 2: −18 to −22 °C; ST 3: −22 to −30 °C; ST 4: −20 to −25 °C), as did B_LTE_90_ (ST 1: −12 to −17 °C; ST 2: −20 to −23 °C; ST 3: −25 to −33 °C; ST 4: −23 to −27 °C). Numerical differences among cultivars and diameter classes were present at individual sampling times, though considerable overlap was observed across the dataset (**Figure 6**). Phloem exotherms (P_LTE) showed a similar temporal pattern across all three thresholds (**Figure 6**). The P_LTE_10_ ranged from approximately −8 to −15 °C at ST 1, declining to −22 to −27 °C at ST 4. The P_LTE_50_ values ranged from approximately −13 to −16 °C at ST 1 to −26 to −32 °C at ST 4, and P_LTE_90_ from approximately −15 to −18 °C at ST 1 to −30 to −35 °C at ST 4. Some variation among cultivar and diameter class combinations was apparent at each sampling time, but no consistent directional pattern was evident across all sampling times (**Figure 6**). Xylem exotherms (X_LTE) spanned the widest absolute temperature range among all LTE parameters measured, with ST means becoming progressively more negative from ST 1 through ST 3–ST 4 across all thresholds (**Figure 7**). Numerically, ‘Prairie Star’ tended to exhibit slightly more negative X_LTE_50_ values than ‘Frontenac’ (approximately −36.6 vs. −35.6 °C, respectively), though the difference was small. Diameter class did not show a consistent or directional influence on X_LTE values across sampling times (**Figure 7**). Bud survival remained high throughout the experiment, ranging from 91.3 to 100.0% across all treatment combinations and sampling times (**Figure 7**). The ST means exceeded 96% at all sampling times, and no meaningful temporal decline was observed. Diameter class had a negligible effect on bud viability (**Figure 7**).

### 3.4 Principal component analysis

**Figure 8:**
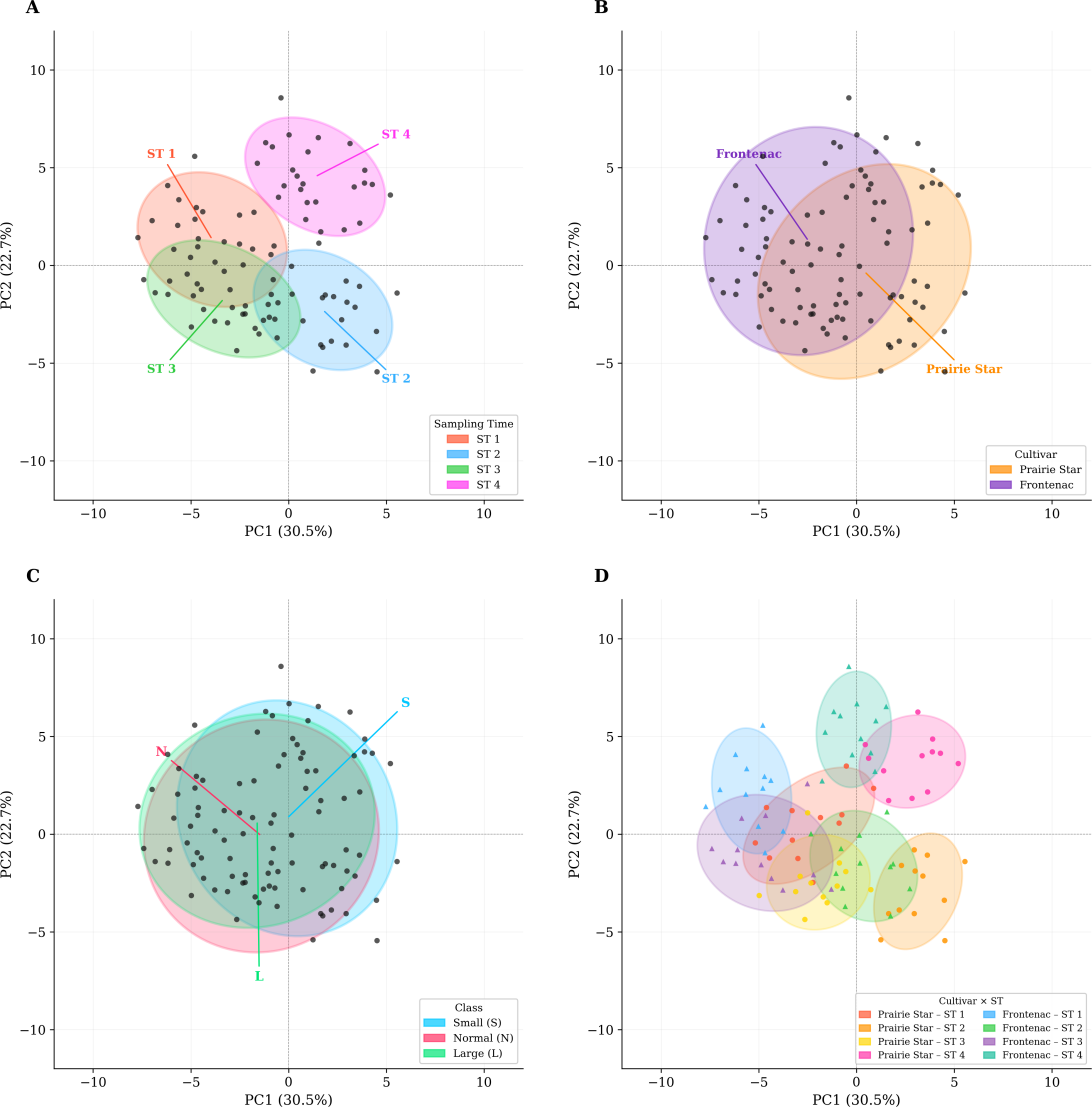
Principal component analysis (PCA) biplot of morphological parameters, shoot color parameters and cane and bud low temperature exotherms in ‘Prairie Star’ and ‘Frontenac’ grapevine genotypes. **(A)** distribution of observations by sampling time (ST 1–4); **(B)** distribution by cultivar (Prairie Star and Frontenac); **(C)** distribution by internode class (S: small, N: normal, L: large); **(D)** combined distribution by cultivar and sampling time. Ellipses represent 95% confidence intervals. PC1 and PC2 explain 30.5% and 22.7% of the total variance, respectively.

The PCA conducted on the complete dataset yielded a two-dimensional solution in which PC1 and PC2 explained 30.5% and 22.7% of the total variance, respectively, accounting for 53.2% of the overall variation (**Figure 8**). When observations were grouped by sampling time, ST 1 and ST 4 were positioned toward opposite ends of the PC1 axis, with their 95% confidence ellipses showing no overlap; ST 2 and ST 3 occupied intermediate positions with partially overlapping ellipses, indicating greater similarity between mid-season sampling periods relative to ST 1 and ST 4 (**Figure 8A**). Grouping by cultivar revealed substantial overlap between the confidence ellipses of ‘Prairie Star’ and ‘Frontenac’ along both principal components, though their centroids were slightly displaced, with ‘Prairie Star’ positioned toward positive and ‘Frontenac’ toward negative PC1 values (**Figure 8B**). When grouped by internode diameter class, the three class ellipses partially overlapped, with the small-class ellipse positioned in the upper-right region, the normal-class ellipse centrally, and the large-class ellipse toward the lower region of the biplot, suggesting a gradient in multivariate shoot characteristics associated with diameter class (**Figure 8C**). In the combined cultivar × sampling time panel, separation was predominantly driven by sampling time along PC1, with the greatest between-cultivar separation observed at ST 1 and ST 4 and greater overlap between cultivars at ST 2 and ST 3 (**Figure 8D**).

### 3.5 Correlation matrix

**Figure 9:**
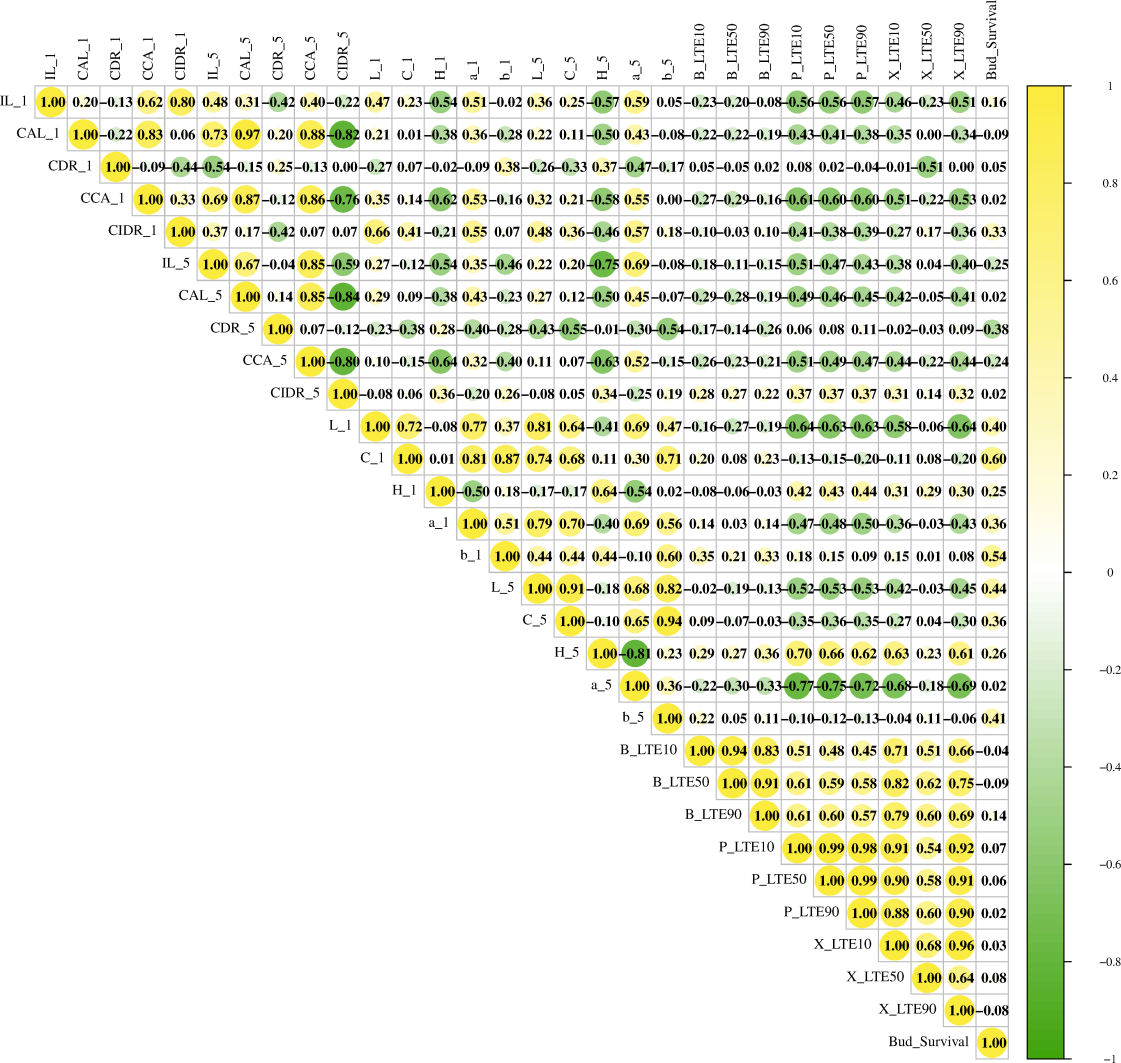
Correlation matrix of morphological parameters (IL_1_, IL_5_, CAL_1_, CAL_5_, CDR_1_, CDR_5_, CCA_1_, CCA_5_, CIDR_1_ and CIDR_5_), shoot color parameters (L*_1_, L*_5_, C*_1_, C*_5_, H°_1_, H°_5_, a*_1_, a*_5_, b*_1_ and b*_5_) and cane and bud low temperature exotherms (B_LTE_10_, B_LTE_50_, B_LTE_90_, P_LTE_10_, P_LTE_50_, P_LTE_90_, X_LTE_10_, X_LTE_50_, X_LTE_90_) and Bud Survival (%) in ‘Prairie Star’ and ‘Frontenac’ grapevine genotypes. Pearson correlation coefficients are shown; color intensity indicates the strength and direction of the correlation (red: positive, blue: negative). Asterisks denote statistical significance: *p<0.05. Abbreviations: CCA_1; cross-sectional area for internode 1, CCA_5; cross-sectional area for internode 5, CAL_1; average diameter for internode 1, CAL_5; average diameter for internode 5, CDR_1; cane diameter ratio for internode 1, CDR_5; cane diameter ratio for internode 5, CIDR_1; cane internode to diameter ratio for internode 1, CIDR_5; cane internode to diameter ratio for internode 5, IL_1; internode length for internode 1, IL_5; internode length for internode 5. B_LTE_10/50/90_; bud LTE_10/50/90_, bud_survival; percent of buds 1 to 6 alive at sampling, P_LTE_10/50/90_; phloem LTE_10/50/90_, X_LTE_10/50/90_; xylem LTE_10/50/90_.

Within-tissue inter-correlations among LTE thresholds were high across all tissue types (**Figure 9**). Phloem exotherm thresholds were the most tightly intercorrelated (P_LTE_10_–P_LTE_50_: r = 0.99; P_LTE_10_–P_LTE_90_: r = 0.98; P_LTE_50_–P_LTE_90_: r = 0.99). Xylem exotherm thresholds also showed strong mutual associations (X_LTE_10_–X_LTE_90_: r = 0.96; X_LTE_10_–X_LTE_50_: r = 0.68; X_LTE_50_–X_LTE_90_: r = 0.64), as did bud exotherms (B_LTE_10_–B_LTE_50_: r = 0.94; B_LTE_10_–B_LTE_90_: r = 0.83; B_LTE_50_–B_LTE_90_: r = 0.91). Cross-tissue associations were moderately positive between bud and phloem exotherms (r = 0.45–0.61), and stronger between phloem and xylem exotherms (P_LTE_10_–X_LTE_10_: r = 0.91; P_LTE_50_–X_LTE_50_: r = 0.91; P_LTE_90_–X_LTE_90_: r = 0.90) (**Figure 9**). Among morphological parameters, CAL_1_ and CAL_5_ were strongly positively correlated (r = 0.97), and CCA_1_ showed strong positive associations with CAL_5_ (r = 0.87) and CCA_5_ (r = 0.86), and a moderate positive association with IL_5_ (r = 0.69) (**Figure 9**). The IL_1_ showed a positive correlation with CCA_1_ (r = 0.62) and CAL_1_ (r = 0.20), while CDR_1_ and CDR_5_ displayed weak or negligible correlations with most other parameters. Within the colorimetric parameters, L_1_ was positively associated with C_1_ (r = 0.72) and a_1_ (r = 0.77), while H°_1_ was negatively correlated with a_1_ (r = −0.50) and b_1_ (r = −0.17). At position 5, L_5_ and C_5_ were strongly positively correlated (r = 0.91), and H°_5_ showed a strong negative association with a_5_ (r = −0.81) (**Figure 9**). Regarding associations between colorimetric and cold hardiness parameters, a_5_ showed the strongest negative correlations with phloem exotherms (P_LTE_10_: r = −0.77; P_LTE_50_: r = −0.75; P_LTE_90_: r = −0.72) and with xylem exotherms (X_LTE_10_: r = −0.68; X_LTE_50_: r = −0.69). The H°_5_ was positively associated with xylem exotherms (X_LTE_10_: r = 0.70; X_LTE_50_: r = 0.66; X_LTE_90_: r = 0.62) and with P_LTE_10_ (r = 0.36). Bud survival showed no strong correlation with any individual parameter; the highest absolute values were recorded with b_1_ (r = 0.54) and L*_5_ (r = 0.44) (**Figure 9**).

### 3.6 Heatmap

**Figure 10:**
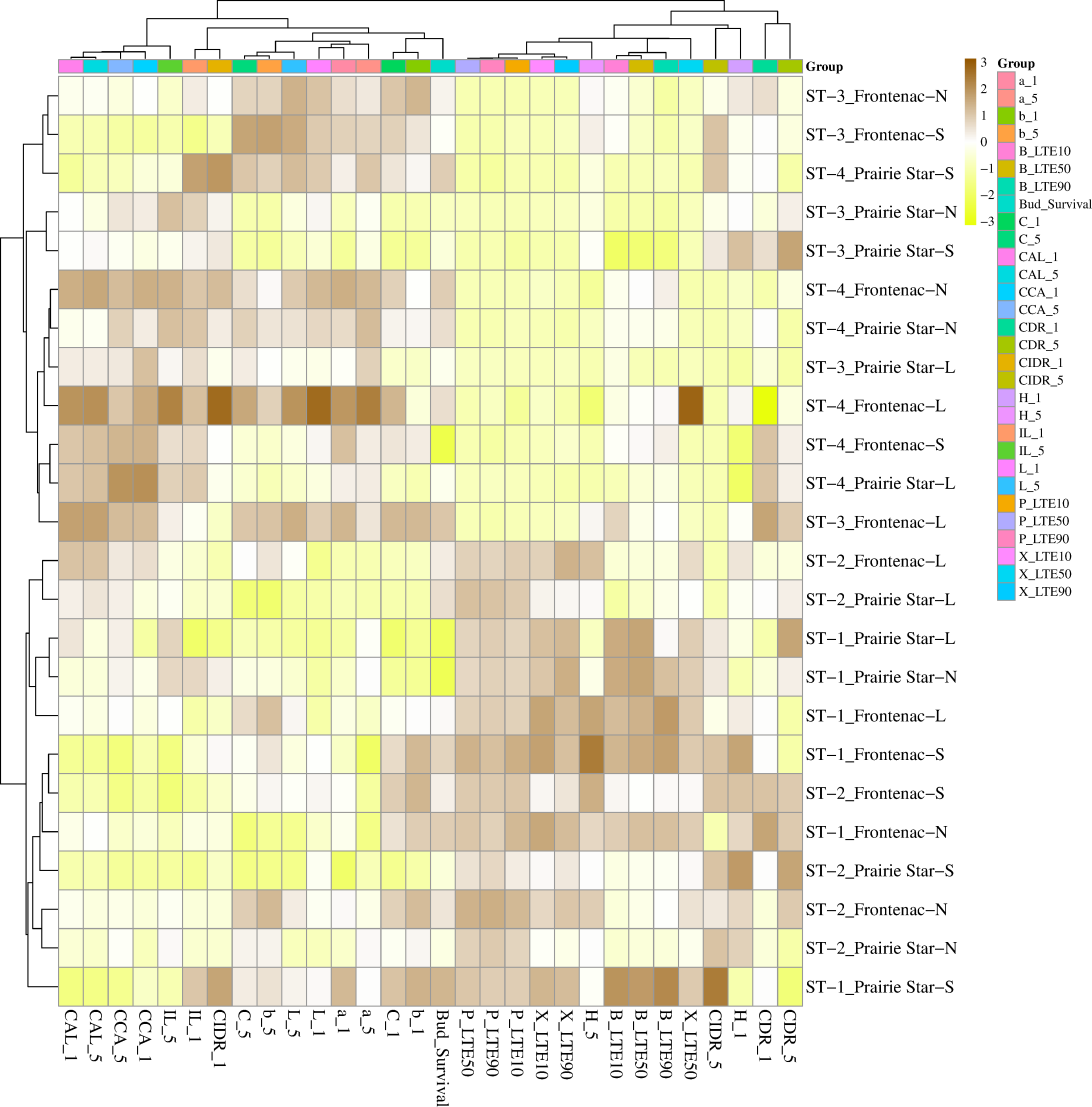
Heatmap of mean values of morphological parameters (IL_1_, IL_5_, CAL_1_, CAL_5_, CDR_1_, CDR_5_, CCA_1_, CCA_5_, CIDR_1_ and CIDR_5_), shoot color parameters (L*_1_, L*_5_, C*_1_, C*_5_, H°_1_, H°_5_, a*_1_, a*_5_, b*_1_ and b*_5_) and cane and bud low temperature exotherms (B_LTE_10_, B_LTE_50_, B_LTE_90_, P_LTE_10_, P_LTE_50_, P_LTE_90_, X_LTE_10_, X_LTE_50_, X_LTE_90_) and Bud Survival (%) in ‘Prairie Star’ and ‘Frontenac’ grapevine genotypes under three internode classes (S: small, N: normal, L: large) across four sampling times (ST 1–4). Abbreviations: CCA_1; cross-sectional area for internode 1, CCA_5; cross-sectional area for internode 5, CAL_1; average diameter for internode 1, CAL_5; average diameter for internode 5, CDR_1; cane diameter ratio for internode 1, CDR_5; cane diameter ratio for internode 5, CIDR_1; cane internode to diameter ratio for internode 1, CIDR_5; cane internode to diameter ratio for internode 5, IL_1; internode length for internode 1, IL_5; internode length for internode 5. B_LTE_10/50/90_; bud LTE_10/50/90_, bud_survival; percent of buds 1 to 6 alive at sampling, P_LTE_10/50/90_; phloem LTE_10/50/90_, X_LTE_10/50/90_; xylem LTE_10/50/90_.

Hierarchical clustering of the 24 treatment combinations across all measured parameters revealed two primary row clusters broadly corresponding to early (ST 1 and ST 2) and late (ST 3 and ST 4) sampling times (**Figure 10**). Late-sampling-time treatments, particularly ST 3 and ST 4 combinations, displayed consistently elevated standardized values for morphological parameters including CAL_1_, CAL_5_, CCA_1_, CCA_5_, and IL_5_, as indicated by intense brown coloration, with the ST-4_Frontenac-L treatment producing the most extreme positive values for these parameters. The ST 1 and ST 2 treatment combinations showed elevated standardized values for LTE parameters across bud, phloem, and xylem tissues, consistent with warmer (less negative) exotherm temperatures at early sampling times; yellow coloration for LTE parameters was concentrated in ST 3 and ST 4 rows, reflecting more negative exotherm values at later periods. Column clustering grouped LTE parameters into a discrete cluster, separate from morphological and colorimetric parameters. The CDR_1_ and CDR_5_ displayed uniformly neutral values across all treatment combinations. Within the colorimetric parameter cluster, a_1_, a_5_, b_1_, and b_5_ showed elevated standardized values at ST 3 and ST 4, particularly for ‘Frontenac’ large-class shoots, while ST 1 and ST 2 rows displayed uniformly low values for these parameters. Hue angle parameters (H_1 and H_5) clustered adjacently to CIDR_5_ and B_LTE parameters. Bud survival values remained consistently neutral across all treatment rows, consistent with the narrow numerical range observed in the raw data. Within the row dendrogram, ‘Prairie Star’ and ‘Frontenac’ treatments at the same sampling time and internode class were generally positioned in adjacent sub-clusters (**Figure 10**).

## 4 Discussion

### 4.1 Evaluation of internode measurements

The results of the present study indicate that sampling time, genotype, and cane diameter class influenced IL_1_, CAL_1_, CDR_1_, CCA_1_, CIDR_1_, IL_5_, CAL_5_, CDR_5_, CCA_5_, and CIDR_5_; however, these effects did not follow a consistently progressive pattern across sampling periods (**Figures 2**, **3**). The IL_1_ ranged approximately 10–20 mm with no consistent temporal trend across sampling times, while CCA_1_ increased from approximately 3,000–5,000 mm^2^ at ST 1 to 10,000–12,500 mm^2^ at ST 4, with considerable intermediate variability (**Figures 2**, **3**). This lack of a uniform trend suggests that observed variation likely reflects sampling variability and differences in cane selection rather than a steady developmental progression. Previous studies have shown that grapevine vegetative growth is strongly influenced by environmental and management factors, including nutrient availability and seasonal conditions (Keller et al., 2001; Petrie et al., 2000). Factors such as temperature variation, water availability, and crop load are known to affect shoot elongation and thickening processes throughout the growing season (Keller et al., 2005). Genotypic differences were evident but remained relatively moderate (**Figure 2**). ‘Prairie Star’ exhibited higher IL_1_ values (≈15–18 mm) compared to ‘Frontenac’ (≈10–14 mm), while at the fifth node, ‘Prairie Star’ (≈95–102 mm) consistently exceeded ‘Frontenac’ (≈58–65 mm) in IL_5_. ‘Frontenac’ showed greater shoot diameter, particularly in CAL_5_ (≈8.9 mm vs. ≈7.4 mm). These differences are consistent with earlier findings emphasizing the role of genetic background in determining vine vigor and structural traits (Grigg, 2017; Dinu et al., 2021). However, the absence of a stable pattern across all sampling times indicates that genotype effects interacted with temporal factors.

Cane diameter class had a clearer effect on diameter- and area-related traits (**Figures 2**, **3**). CCA_1_ values were consistently higher in large-class shoots than in normal and small classes, following an L > N > S gradient, which was also reflected in CAL_1_ (≈10.6 mm, 8.6 mm, and 6.9 mm for large, normal, and small classes, respectively) and CAL_5_ (≈10.2 mm, 8.2 mm, and 6.8 mm) (**Figure 2**). These findings are in line with established knowledge that thicker shoots are associated with greater secondary growth and increased vascular development. Previous studies have shown that basal portions of grapevine shoots undergo greater radial growth and lignification (Schubert et al., 1999; May, 2000; Zhang et al., 2003; Köse and Güleryüz, 2009; McLoughlin et al., 2011; Torregrosa et al., 2021), which is consistent with the larger cross-sectional area observed in the present study (**Figure 3**). In contrast, internode length parameters (IL_1_ and IL_5_) did not display a consistent ranking among classes or sampling times (**Figure 2**). Although IL_5_ values (≈58–105 mm) were substantially higher than IL_1_ values (≈10–20 mm), both parameters showed fluctuations rather than a clear class-dependent or temporal gradient, supporting the view that internode elongation was more sensitive to short-term environmental and physiological conditions than to structural traits such as diameter or cross-sectional area (Lebon et al., 2004; Ritter et al., 2024). The CDR_1_ and CDR_5_ values remained within a narrow range (0.95–1.26 and 1.0–1.2, respectively), indicating relative stability across genotypes, classes, and sampling times (**Figure 2**). Similarly, CIDR_1_ (≈0.03–0.24) and CIDR_5_ (≈0.10–0.26) exhibited variability without a consistent temporal or class-related trend (**Figure 3**). The stability of these ratio-based parameters suggests that proportional relationships between shoot dimensions were largely maintained despite fluctuations in absolute values.

### 4.2 Evaluation of cane color measurements

The results of the present study highlighted the intricate interplay between sampling time, genotype, and internode diameter class that influenced the color parameters of grapevine canes (**Figures 4**, **5**). These findings align with the existing body of knowledge in viticulture and vine cane physiology, contributing valuable insights into the dynamic processes governing shoot coloration in grapevines (Keller, 2010). The observed changes in color parameters across different sampling times are consistent with the well-documented developmental patterns of grapevine shoots (Palliotti et al., 2014). The L_1_ values showed a pronounced minimum at ST 2 (mean: 26.3) and a maximum at ST 4 (mean: 45.0), while a_1_ rose from a mean of 6.3 at ST 1 to 14.1 at ST 4, representing the most pronounced temporal increase of all measured color parameters (**Figure 5**). Hue angle exhibited a consistent decline at both node positions, with H°_1_ decreasing from 66.2° at ST 1 to 53.9° at ST 4 and H°_5_ from 70.4° to 52.1°, indicating a progressive shift toward redder and browner tones as the season advanced (**Figure 4**). Notably, color parameters at position 5 consistently differed from those at position 1 across sampling times; for instance, C_5_ reached its peak at ST 3 (31.8) rather than ST 4, and a_5_ values were substantially higher than a*_1_ at ST 4 (18.4 vs. 14.1), suggesting that distal internode positions may undergo earlier or more intense pigmentation changes than basal positions, possibly reflecting greater light exposure or more advanced lignification at the shoot tip (Lebon et al., 2004; Gambetta et al., 2021) (**Figures 4**, **5**). These trends are consistent with previous studies that have documented the accumulation of pigments and lignification processes over the growing season (Gambetta et al., 2021). Increased sunlight exposure following leaf fall can trigger the accumulation of phenolic compounds and pigment production, contributing to the observed changes in cane coloration (Fang et al., 2015; Gambetta et al., 2021).

Genotypic differences were evident in the colorimetric data (**Figures 4**, **5**), with ‘Frontenac’ exhibiting higher a_5_ (13.0 vs. 11.1), b_5_ (25.0 vs. 23.2), and C_5_ (27.9 vs. 25.6) values compared to ‘Prairie Star’, while cultivar differences in L and H° were minimal at both node positions. These differences were attributed to the inherent genetic variations between the two genotypes, which influence various aspects of shoot development, pigmentation, and overall coloration (Tikhonov et al., 2017). The consistent direction of these genotypic differences across sampling times and diameter classes suggested that colorimetric traits at the distal internode position, particularly a_5_ and C_5_, may be under a degree of genetic control, a consideration that warrants attention in interpreting the broader applicability of these findings across cultivars (Tikhonov et al., 2017; Keller, 2010). Furthermore, the internode diameter class had a measurable impact on several color parameters, with small-class shoots tending to exhibit higher H° values (H°_1_: 63.1°; H°_5_: 63.0°) compared to large-class shoots (H°_1_: 57.7°; H°_5_: 58.6°), while large-class shoots recorded the highest a*_1_ means (9.4 vs. 8.7 for normal and 7.9 for small class), and these differences were interpreted in the context of shoot thickness and maturation stage rather than treated as standalone descriptive findings (**Figures 4**, **5**). This trend is in line with previous studies documenting the influence of shoot thickness and maturation stage on coloration characteristics (Lebon et al., 2004). Considering the limited studies previously conducted on this subject, further research is warranted to elucidate the underlying mechanisms driving the observed patterns across a broader range of grape cultivars and environmental conditions.

### 4.3 Evaluation of cold hardiness in buds and canes

The evaluation of cold hardiness in buds and canes provided valuable insights into the effects of sampling time, genotype, and cane diameter class on critical temperatures related to bud and cane low temperature exotherms and bud survival (**Figures 6**, **7**). The sampling time played a crucial role in modulating cold hardiness parameters, with ST 3 and ST 4 generally exhibiting more negative LTE values compared to ST 1 and ST 2. The B_LTE_10_ means at ST 2, ST 3, and ST 4 (−18.15, −18.18, and −18.05 °C, respectively) were substantially more negative than at ST 1 (−10.42 °C), while phloem exotherms showed the sharpest transition between ST 2 and ST 3, with P_LTE_10_ means shifting from −11.97 °C to −20.75 °C (**Figure 6**). Bud low temperature exotherm values at the 50% threshold (B_LTE_50_) ranged from −10.2 °C (‘Prairie Star’, small class, ST 1) to −26.1 °C (‘Prairie Star’, small class, ST 3), reflecting a substantial increase in bud cold hardiness across the sampling period. The most notable transition occurred between ST 1 (mean: −11.9 °C) and ST 2 (mean: −19.7 °C), indicating that the majority of bud cold acclimation was achieved rapidly during the early dormancy period, after which ST 3 (mean: −22.2 °C) and ST 4 (mean: −19.4 °C) produced broadly comparable and more negative values. The slight reversal observed between ST 3 and ST 4 may reflect a partial de-acclimation response associated with fluctuating late-season temperatures, a phenomenon previously documented in dormant grapevine buds exposed to transient warming events (Londo and Kovaleski, 2017). ‘Prairie Star’ recorded consistently more negative B_LTE_50_ values than ‘Frontenac’ across all sampling times, suggesting a genotypic advantage in bud cold hardiness that aligns with the known cold tolerance characteristics of this cultivar. This temporal pattern is consistent with the well-established pattern of cold acclimation in grapevines, where hardiness gradually increases as vines transition into dormancy and experience prolonged exposure to low temperatures. De-acclimation occurs when plants are exposed to transient warming periods during mid- or late winter, triggering a partial loss of previously acquired cold tolerance through resumption of metabolic activity, membrane restructuring, and reduction in cryoprotectant concentrations; this process can render vines temporarily more susceptible to subsequent frost events even after full hardening has been achieved (Kovaleski and Londo, 2019; Fennell, 2004). The observed higher hardiness values at later sampling times likely reflect the physiological and biochemical changes associated with cold acclimation, such as the accumulation of cryoprotectants, soluble sugars, and specific amino acids, modification of membrane lipid composition, and reduction in tissue water content, all of which are critical for surviving low-temperature conditions (Fennell, 2004; Rende et al., 2018; Keller, 2010; Kaya, 2020a, 2020b). It has been reported that as temperatures gradually decrease from autumn into winter, grapevines undergo significant metabolic adjustments to enhance their cold tolerance, including increasingthe proportion of unsaturated fatty acids to maintain membrane fluidity (Fennell, 2004; Rende et al., 2018). These findings are consistent with the work of Thomashow (1999) and Guy (1990), who demonstrated that extended exposure to cold periods enhances freezing tolerance in plants. Levitt (1980) further confirmed that prolonged cold exposure increases cold hardiness through a comprehensive examination of plant responses to environmental stresses.

Genotypic differences in cold hardiness were evident, with ‘Prairie Star’ consistently recording more negative xylem exotherm values across all thresholds (X_LTE_10_: −32.6 °C; X_LTE_50_: −36.58 °C; X_LTE_90_: −40.40 °C) compared to ‘Frontenac’ (X_LTE_10_: −31.79 °C; X_LTE_50_: −35.57 °C; X_LTE_90_: −39.23 °C), as well as slightly more negative B_LTE_10_ values (−17.03 vs. −15.38 °C) (**Figure 7**). Critically, the direction of these genotypic differences was maintained across all four sampling times and across diameter classes, suggesting that the between-cultivar contrast in cold hardiness reflects a stable genotypic signal rather than an interaction-dependent or environmentally contingent effect. In the context of a single-season study, the consistency of this genotypic ranking across repeated temporal observations strengthens the inference that the observed differences are attributable primarily to genetic background rather than to year-specific environmental conditions, and thereby supports the broader generalizability of the cultivar comparison made here (Kovaleski and Londo, 2019; Keller, 2010). These findings align with previous studies highlighting the influence of genetic factors on cold hardiness variability among grapevine cultivars (Londo and Kovaleski, 2017). Kovaleski and Londo (2019) emphasized that genetic makeup is a fundamental determinant of a grapevine’s capacity to acclimate to cold temperatures, while Keller (2010) highlighted the substantial variability in cold hardiness among different cultivars attributable to underlying genetic diversity. The higher xylem hardiness observed in ‘Prairie Star’ suggests that this cultivar has developed more robust physiological and biochemical mechanisms to cope with cold stress, including potentially enhanced accumulation of cryoprotectants and more efficient modification of membrane lipid composition (Dami and Zhang, 2023). Bud survival rates remained consistently high throughout the experiment, ranging from 91.3% to 100.0%, with all ST means above 96% and no marked temporal decline detected (**Figure 6**). ‘Frontenac’ (97.46%) demonstrated marginally higher bud survival rates than ‘Prairie Star’ (96.16%), and internode class had a negligible effect. These findings contrast with conditions in which more severe winter temperatures would be expected to produce greater variation in bud survival, as documented in prior years with harsher winters (Ferguson et al., 2014). Cane diameter class had a limited but measurable influence on specific cold hardiness parameters, particularly xylem exotherms, with small-class shoots tending toward marginally more negative LTE values. Previous studies have reported conflicting results regarding the relationship between cane diameter and cold hardiness, with some suggesting a positive correlation (Dami et al., 2016; Todaro and Dami, 2017; Kaya et al., 2024), while others report no significant association (Ren et al., 2021), highlighting the complexity of the underlying mechanisms and the potential interplay with genotype and environmental conditions.

### 4.4 General evaluation

The PCA results revealed clear separation among sampling times along PC1 and PC2, which together explained 53.2% of the total variance (**Figure 8**). ST 1 and ST 4 occupied opposite ends of the PC1 axis with non-overlapping confidence ellipses, while ST 2 and ST 3 showed partial intersection, indicating greater similarity between mid-season sampling periods (**Figure 8A**). These temporal patterns in multivariate space align with existing literature suggesting that various factors interact and contribute to cold hardiness phenomena in grapevines, with temperature fluctuations, day length, and dormancy stage playing crucial roles (Fennell, 2004). The high degree of overlap between cultivar ellipses (**Figure 8B**) is consistent with the observation that closely related genotypes often share commonalities in their physiological responses (Kaya, 2020b; Kaya et al., 2024), while the centroid displacement between ‘Prairie Star’ and ‘Frontenac’ along PC1 reflects the genotypic differences documented in cold hardiness and morphological parameters. This centroid displacement, though modest, is consistent with the persistent between-cultivar differences observed across all measured parameter groups, and may represent the multivariate expression of a genetically determined phenotypic profile that is stable across seasonal conditions, a pattern that, despite the single-season design of this study, supports cautious generalization of the cultivar-level findings (Kovaleski and Londo, 2019; Keller, 2010). Internode class ellipses showed a spatial gradient from small-class in the upper-right to large-class in the lower region of the biplot (**Figure 8C**), indicating that cane diameter class contributes to observed data patterns alongside other factors such as carbohydrate reserves (Striegler et al., 2000). Hierarchical clustering in the heatmap analysis revealed two primary treatment groups corresponding to early (ST 1 and ST 2) and late (ST 3 and ST 4) sampling times (**Figure 10**). Late sampling time treatments displayed consistently elevated values for morphological parameters (CAL_1_, CAL_5_, CCA_1_, CCA_5_, IL_5_) and more negative LTE values, while ST-1 and ST-2 combinations showed warmer exotherm temperatures across all tissues. The formation of distinct clusters based on the combination of sampling time, genotype, and internode class further highlights the complex interplay of factors influencing cold hardiness in grapevines, aligning with previous studies that have emphasized the importance of considering multiple factors in understanding cold hardiness responses (Ferguson et al., 2014; Kaya et al., 2024).

The correlation matrix results revealed several significant relationships among the parameters measured in this study, highlighting the complex interplay of factors influencing cold hardiness in grapevines (**Figure 9**). Strong positive correlations were observed within LTE parameter groups, with phloem exotherm thresholds showing near-perfect inter-correlations (P_LTE_10_–P_LTE_50_: r = 0.99; P_LTE_50_–P_LTE_90_: r = 0.99), xylem exotherms displaying very high associations (X_LTE_10_–X_LTE_90_: r = 0.96), and bud exotherms exhibiting strong mutual associations (B_LTE_10_–B_LTE_50_: r = 0.94; B_LTE_50_–B_LTE_90_: r = 0.91). Strong positive cross-tissue correlations were also observed between phloem and xylem exotherms (P_LTE_10_–X_LTE_10_: r = 0.91; P_LTE_50_–X_LTE_50_: r = 0.91; P_LTE_90_–X_LTE_90_: r = 0.90), consistent with previous research reporting a strong positive correlation between cane and bud cold hardiness in several grapevine cultivars (Ferguson et al., 2014). This was not surprising, as canes and buds undergo similar physiological changes during cold acclimation, such as increased accumulation of soluble sugars and dehydrins (Rende et al., 2018). The strong negative correlations observed between a_5_ and phloem exotherms (P_LTE_10_: r = −0.77; P_LTE_50_: r = −0.75; P_LTE_90_: r = −0.72) and xylem exotherms (X_LTE_10_: r = −0.68; X_LTE_50_: r = −0.69) indicated that higher red chromatic intensity at the fifth node position was associated with more negative LTE values, suggesting a physiological link between shoot pigmentation and cold hardening (**Figure 9**). Similarly, H°_5_ was positively correlated with xylem exotherms (X_LTE_10_: r = 0.70; X_LTE_50_: r = 0.66; X_LTE_90_: r = 0.62), meaning that shoots with higher hue angle, that is, less red coloration, corresponded to less negative exotherm temperatures. These findings are consistent with studies in model plant systems showing that anthocyanin accumulation contributes to cold acclimation by limiting oxidative stress and photoinhibition under low-temperature conditions (Catalá et al., 2011). Among morphological parameters, CAL_1_–CAL_5_ (r = 0.97) and CCA_1_–CAL_5_ (r = 0.87) were the strongest associations, while CDR_1_ and CDR_5_ displayed negligible correlations with most parameters. Although internode length has been associated with cold hardiness indicators in previous studies, with shorter internodes attributed to higher concentrations of carbohydrates and biochemical compounds in compact nodes (Striegler et al., 2000; Fennell, 2004; Levitt, 1980; Kaya, 2020a), no internode length data were included in the correlation analysis of the present study, and therefore this relationship could not be directly evaluated here. Additionally, C_1_ showed relatively notable positive correlations with bud survival (r ≈ 0.60), which, while not among the strongest associations observed, may reflect the broader relationship between shoot maturation and pigmentation intensity, and warrants further investigation (**Figure 9**). Bud survival showed no strong correlation with any individual parameter, with the highest absolute values recorded against b_1_ (r = 0.54) and L_5_ (r = 0.44).

## 5 Conclusion

This study demonstrated that sampling time was the dominant source of variation across morphological, colorimetric, and cold hardiness parameters in ‘Prairie Star’ and ‘Frontenac’ grapevines, with the most pronounced acclimation occurring between ST 1 and ST 2. Morphological traits generally tended to increase toward later sampling times, while shoot coloration shifted toward darker and redder–brown tones, consistent with lignification. Cold hardiness of bud, phloem, and xylem tissues increased most noticeably between ST 1 and ST 2, indicating a key period of acclimation, whereas bud survival remained high across treatments. Genotypic differences were present but moderate: ‘Prairie Star’ tended to exhibit longer internodes and slightly greater xylem cold hardiness, whereas ‘Frontenac’ showed larger cane diameters and marginally higher bud survival. Internode class mainly influenced morphological traits, with large-class shoots having greater diameter and cross-sectional area, while small-class shoots showed relatively higher pith-to-diameter ratios and slightly more negative LTE values. Correlation analysis indicated that shoot color parameters at the fifth internode, particularly a and H°, were among the strongest non-destructive indicators of phloem and xylem cold hardiness, underscoring the practical value of distal internode colorimetric assessments for field-based cold hardiness screening. The association between C_1_ and bud survival further suggests that chroma at the first internode position may provide supplementary information on bud viability, though this relationship requires validation across broader conditions. These relationships suggest potential for practical use in field-based assessments. Future studies should validate these findings across additional cultivars and environments, to further investigate the physiological basis of these relationships, and evaluate their applicability in developing non-destructive screening approaches and predictive models for cold hardiness.

## Data Availability

The datasets supporting the findings of this study are stored in the authors’ institutional archives and may contain personal research records. These data are available from the corresponding authors upon reasonable request, in accordance with applicable privacy and institutional regulations.

## Glossary

CCA1: cross-sectional area for internode 1
CCA5: cross-sectional area for internode 5
CAL1: average diameter for internode 1
CAL5: average diameter for internode 5
CDR1: cane diameter ratio for internode 1
CDR5: cane diameter ratio for internode 5
CIDR1: cane internode to diameter ratio for internode 1
CIDR5: cane internode to diameter ratio for internode 5
IL1: internode length for internode 1
IL5: internode length for internode 5
a1: a* (green-red) for internode 1
a5: a* (green-red) for internode 5
b1: b* (blue-yellow) for internode 1
b5: b* (blue-yellow) for internode 5
C1: C* (chroma) for internode 1
C5: C* (chroma) for internode 5
H1: h° (hue angle) for internode 1
H5: h° (hue angle) for internode 5
L1: L* (lightness) for internode 1
L5: L* (lightness) for internode 5
B_LTE10/50/90: bud LTE10/50/90
bud_survival: percent of buds 1 to 6 alive at sampling
P_LTE10/50/90: phloem LTE10/50/90
X_LTE10/50/90: xylem LTE10/50/90.
